# Exploring the Interactive Role of Parathyroid Hormone and Sodium Intake in Inducing Cardiac Hypertrophy in Rats: A Novel Study

**DOI:** 10.7759/cureus.45154

**Published:** 2023-09-13

**Authors:** Quan Liang, Shiqiong Huang, Jianhang Wei, Wenbin Deng, Lihua Li

**Affiliations:** 1 Department of Gerontology, The First Affiliated Hospital of Dali University, Dali, CHN

**Keywords:** renin-angiotensin-aldosterone system, salt, myocardial fibrosis, left ventricular hypertrophy, parathyroid hormone

## Abstract

Background and objectives

Previous research has suggested that hyperparathyroidism and excessive salt intake may contribute to the development of cardiac hypertrophy. This study aimed to investigate the relationship and underlying mechanisms between parathyroid hormone (PTH) and salt intake in the development of left ventricular hypertrophy (LVH). Additionally, the study sought to determine whether captopril intervention could reduce the impact of sustained PTH stimulation and excessive salt intake on LVH.

Methodology

We employed 40 eight-week-old male Sprague-Dawley rats, which were randomly assigned to eight groups: a sham group, a PTH group, a low-salt group (0.6% NaCl), a high-salt group (8% NaCl), a PTH + low-salt group, a PTH + high-salt group, a PTH + low salt + captopril group, and a PTH + high salt + captopril group. The rats were continuously infused with recombinant PTH (1-34) (2 pmol/kg per hour) via an osmotic pump for two weeks and were administered varying concentrations of saline for gavage over two weeks, according to their group. We monitored changes in blood pressure, measured heart weight, left ventricular wall thickness, and myocardial histological morphology, and assessed the relative expression of type III collagen.

Results

The PTH + high-salt group displayed a significant increase in blood pressure, heart weight, and left ventricular posterior wall thickness (*P*＜0.05), in addition to myocardial cell hypertrophy and increased Col III expression (*P*＜0.05), compared to other groups. Captopril intervention significantly reduced blood pressure (*P*＜0.05), ameliorated myocardial tissue morphology changes, and significantly decreased Col III expression (*P*＜0.05) but did not entirely reverse the increase in heart weight and left ventricular posterior wall thickness (*P*＞0.05).

Conclusions

Our findings suggest that the co-intervention of PTH and high salt can lead to an increase in blood pressure, heart weight, myocardial cell hypertrophy, LVH, and myocardial fibrosis levels in Sprague-Dawley rats. Captopril intervention can lower blood pressure and alleviate pathological myocardial tissue changes and myocardial fibrosis but cannot completely reverse LVH.

## Introduction

Parathyroid hormone (PTH), a polypeptide hormone, is primarily secreted by the parathyroid glands. Its main function is to regulate calcium and phosphorus metabolism within the body, predominantly targeting the bones and kidneys. Many patients with chronic kidney disease (CKD) often exhibit severe secondary hyperparathyroidism. Recent research has indicated that PTH can also profoundly impact the cardiovascular system, potentially leading to hypertension, left ventricular hypertrophy (LVH), heart failure, and other cardiovascular diseases [1,2].

The renin-angiotensin-aldosterone system (RAAS) is known to play a pivotal role in the development of cardiovascular and renal diseases. Angiotensin II (Ang II), a system component, can enhance myocardial contractility and stimulate myocardial cell hypertrophy and fibrosis. Conversely, angiotensin-converting enzyme inhibitors (ACEI) such as captopril have been clinically proven to lower blood pressure and improve the prognosis of cardiovascular diseases. Interestingly, studies have discovered an interaction between PTH and RAAS, which may contribute to the pathogenesis of LVH [3]. Furthermore, high salt intake has been identified as a significant factor contributing to the global increase in hypertension incidence. It is also closely associated with the outcomes of LVH, heart failure, and other cardiovascular diseases [4,5]. The regulation of RAAS is inherently linked to these outcomes.

This study is designed to investigate the combined impact of PTH and salt on blood pressure, LVH, and myocardial fibrosis in rats. The objective is to ascertain if administering captopril can alleviate the harmful effects of sustained PTH and salt stimulation on the heart. The findings of this research will shed light on the role and potential mechanism of the interaction between PTH and salt in the development of LVH.

## Materials and methods

Experimental animals and grouping

We procured 40 male Sprague-Dawley rats, aged eight weeks and weighing between 300 and 350 g, from Hunan Slack Jingda Experimental Animal Co., Ltd. (license number SCXK (Xiang) 2019-0004). The rats were accommodated in the animal observation room of the Experimental Animal Science and Technology Center at Dali University's Xiaguan Campus in Yunnan Province. The environment was well-ventilated and quiet, with a temperature of approximately 23 ± 1 ℃, humidity between 40% and 60%, and a 12-hour light exposure cycle. The rats had unrestricted access to water and food.

The rats were randomly segregated into eight groups: sham group, PTH group, low-salt group (0.6% NaCl), high-salt group (8% NaCl), PTH + low-salt group, PTH + high-salt group, PTH + low salt + captopril group, and PTH + high salt + captopril group. Each group comprised five rats. One rat died during the feeding process in the PTH + low salt + captopril group, so this group had only four rats.

All procedures complied with the animal experiment ethics requirements of Dali University and adhered to the Reduce, Reuse, Recycle (3R) principle (approval numbers 2020-SL-02 and 2020-PZ-02).

Experimental reagents and instruments

For our experimental procedures, recombinant mouse PTH1-34 (1 mg/vial, catalog no. 52232-67-4, Tocris Bioscience, Bristol, UK) and Captopril (10 g/bottle, Catalog no. 62571-86-2, Solarbio Science and Technology, Beijing, China) were utilized. A universal immunohistochemical detection kit for mouse/rabbit (Catalog no. KIHC-5, Proteintech, Rosemont, IL, USA), anti-type III collagen antibody (Catalog no. ab6310, Abcam, Cambridge, UK), hematoxylin solution, and 0.5% eosin ethanol solution (Hefei Bomei Biotechnology, Tianjin, China) were also employed. Furthermore, an implantable capsule osmotic pump (model no. 1002, DURECT Corporation, Cupertino, CA, USA), a noninvasive blood pressure measurement system for small animals (Xinruan Information Technology, Shanghai, China), and an optical microscopy photographic system (Olympus, Tokyo, Japan) were used.

Pretreatment of the capsule osmotic pump

For the preliminary preparation of the osmotic pump capsule, recombinant mouse PTH (1-34) was dissolved in sterile water for injection at a concentration of 1 mg/10 mL. This solution was then introduced into the osmotic pump capsule. For the control groups, which included the sham and low- and high-salt groups, the osmotic pump capsule was filled with sterile water for injection. Conversely, for the remaining experimental groups, the capsule was filled with the prepared solution of recombinant mouse PTH (1-34). To ensure a stable release rate, the capsule pump was soaked in physiological saline at 36.5 ℃ for six hours before implantation.

Surgical and pharmacological interventions

Before surgical and pharmacological procedures were initiated, the rats underwent an eight-hour fasting period. The rats were weighed before the administration of anesthesia. Anesthesia was induced through an intraperitoneal injection of 5% chloral hydrate, with a dosage range of 0.5-1 mL/100 g. Following the onset of anesthesia, the rats were immobilized, their skin was prepared, and the surgical site was sterilized. A transverse incision measuring 0.5-1 cm was made below the posterior part of the scapula using a surgical scalpel and fine scissors. The outlet of the capsule pump was then inserted subcutaneously with the outlet oriented inward. The incision was subsequently sutured and disinfected using iodine. The capsule pump was programmed to continuously infuse rat recombinant PTH (1-34) at 2 pmol/kg per hour for two weeks. The rats were given gastric lavage using a gastric lavage needle 16 with different concentrations of saline (0.6% NaCl or 8% NaCl) or captopril (10 mg/kg per day). The lavage volume used was 10-20 mL/kg per day, depending on the experimental group.

Noninvasive blood pressure measurement

Noninvasive blood pressure measurements were conducted on rats before drug intervention and again two weeks postintervention. These measurements were systematically taken between 7 am and 11 am.

For the procedure, the rats were securely placed in a restrainer, and a pulse pressure band was strategically positioned on the upper third of the rat's tail. The highly sensitive pulse transducer's surface was meticulously aligned with the ventral side of the middle third of the tail. To facilitate accurate measurement, the rat's tail was heated to a consistent temperature of 33 °C. Once a stable pulse wave was achieved, systolic, diastolic, and mean arterial pressure measurements were taken. This process was repeated five times consecutively, with the lowest and highest values being disregarded. The remaining three blood pressure data points were then averaged and recorded.

Measurement of cardiac indicators

Rats were anesthetized and sacrificed. The process of gauging cardiac indices necessitated the extraction and meticulous cleaning of the heart from each respective group. The heart weight (HW) was ascertained utilizing a balance with a precision of 0.01 g. The thickness of the left ventricular posterior wall (LVPWT) was measured employing a Vernier caliper, offering a precision of 0.02 mm. The heart mass index (HMI) was computed using the formula HW (mg)/BW (g) = HMI (mg/g).

Hematoxylin and eosin staining of myocardial tissue

Tissue samples from rats' left ventricular apex were procured for hematoxylin-eosin (HE) staining of myocardial tissues. The staining procedure was executed using a solution composed of hematoxylin and eosin. Subsequently, morphological alterations within the myocardial tissues were meticulously observed under a microscope.

Immunohistochemistry

After antigen retrieval on rat myocardial tissue sections for immunohistochemistry, the sections were treated with 3% H_2_O_2_. This was done to inactivate any endogenous peroxidases and was carried out at room temperature for 10 minutes. Following this, the sections were thoroughly washed with saline.

To block any nonspecific binding, the sections were treated with 5% goat serum for one hour. Subsequently, a diluted anti-type III collagen antibody (ab6310, Abcam) was prepared at a ratio of 1:100 and added to the sections. This was then incubated at a temperature of 37 ℃ for one hour. Post incubation, the sections were once again washed with saline. In the next step, 50-100 microL of anti-mouse/rabbit marker (KIHC-5, Proteintech) was added to the sections and incubated at a temperature of 37 ℃ for 30 minutes. In the final steps of this procedure, the sections were stained with 3,3'-diaminobenzidine (DAB) and counterstained with hematoxylin. This was followed by a process of dehydration through an alcohol gradient. The sections were sealed with Neutral Balsam and prepared for observation under a microscope.

Statistical analysis

Statistical analysis was performed on the data using SPSS version 25.0 (IBM Corp., Armonk, NY, USA), and graphs were plotted using GraphPad Prism 8.0 (GraphPad Software, Boston, MA, USA). Measurement data were expressed as mean ± standard deviation, and statistical analysis was conducted using independent samples *t*-test and analysis of variance. A *P*-value of <0.05 was considered statistically significant.

## Results

The impact of PTH and various salt intake concentrations on rat blood pressure

In this experiment, we investigated the effects of different salt concentrations and PTH on the blood pressure of rats. The results indicated that the blood pressure of the PTH + high-salt group significantly increased, which could be reduced to varying degrees after intervention with captopril. Specifically, for systolic blood pressure (SBP), both the PTH + high-salt group and the PTH + high salt + captopril group showed an increase after the intervention and were significantly higher than the sham group. For diastolic blood pressure (DBP), the PTH + high-salt group increased after the intervention and was significantly higher than the sham group, while the PTH group was significantly lower. For mean arterial pressure (MAP), the PTH + high salt + captopril group increased after the intervention and was significantly higher than the sham group. The results of this experiment suggest that both PTH and the intake of different salts impact the blood pressure of rats (Table 1).

**Table 1:** Comparison of blood pressure in different rat groups (mmHg) ^☆^A statistically significant difference compared to the PTH + high-salt group after the intervention, with *P *< 0.05. ^#^A statistically significant difference compared to the sham group after the intervention, with *P* < 0.05. *A statistically significant difference compared to pre-intervention, with *P* < 0.05. ^△^A statistically significant difference compared to the PTH + low-salt group after the intervention, with *P* < 0.05. PTH, parathyroid hormone

Groups	*N*	Systolic blood pressure		Diastolic blood pressure		Mean blood pressure	
		Before intervention	After intervention	Before intervention	After intervention	Before intervention	After intervention
Sham group	5	102.15 ± 1.78	102.96 ± 2.21	86.68 ± 3.71	90.54 ± 2.40	91.84 ± 2.90	94.68 ± 2.25
PTH group	5	97.47 ± 4.06	101.91 ± 12.00^☆^	77.00 ± 9.89	78.58 ± 5.02^#^^☆^	83.83 ± 7.93	86.35 ± 7.11^☆^
Low-salt group	5	101.36 ± 11.26	103.86 ± 4.57^☆^	85.55 ± 8.49	81.54 ± 2.24^☆^	90.82 ± 9.41	88.98 ± 2.97^☆^
High-salt group	5	108.23 ± 13.44	112.60 ± 10.70^☆^	89.14 ± 15.68	85.63 ± 3.22^☆^	95.50 ± 14.81	94.62 ± 4.41^☆^
PTH + low-salt group	5	105.36 ± 7.83	108.02 ± 7.91	90.33 ± 8.74	88.07 ± 13.85	95.34 ± 8.28	94.72 ± 11.83
PTH + high-salt group	5	112.04 ± 4.66	126.90 ± 7.26^*#^^△^	98.18 ± 4.58	105.17 ± 7.81^#^^△^	102.80 ± 4.54	112.41 ± 6.91^#^^△^
PTH + low salt + captopril group	4	107.25 ± 4.94	103.48 ± 4.06^☆^	92.98 ± 6.04	92.12 ± 4.13	97.74 ± 5.64	95.91 ± 3.89^☆^
PTH + high salt + captopril group	5	107.43 ± 5.28	114.57 ± 8.75^*#^^☆^	86.60 ± 7.10	96.46 ± 7.76^*^	93.54 ± 6.33	102.49 ± 7.78^*^

The impact of PTH and different salt intake concentrations on rat HW, HMI, and LVPWT

In this experiment, we investigated the effects of different salt concentrations and PTH on the HW, HMI, and LVPWT of rat myocardial tissue. The results indicated that the HW of rats in the PTH + high-salt group was significantly heavier than that of the sham group, PTH group, low-salt group, and high-salt group (*P *< 0.05). As for HMI, the HMI of the PTH and low-salt groups was significantly lower than that of the PTH + high-salt groups (*P *< 0.05). Regarding LVPWT, the LVPWT of the PTH + high-salt group was significantly thicker compared to the sham and low-salt groups (*P *< 0.05). The specific data can be seen in Table 2.

**Table 2:** Cardiac measurement indices of rats in each group. ^#^A statistically significant difference compared to the PTH + high-salt group after the intervention, with *P* < 0.05. *A statistically significant difference compared to the sham group after the intervention, with *P* < 0.05. BW, body weight; HW, heart weight; HMI, heart mass index; LVPWT, left ventricular posterior wall thickness; PTH, parathyroid hormone

Groups	N	BW (g)	HW (mg)	HMI (mg/g)	LVPWT (mm)
Sham group	5	444.40 ± 51.90	1368.80 ± 104.12	3.13 ± 0.13	2.97 ± 0.51
PTH group	5	375.67 ± 13.58	1234.00 ± 112.58^#^	3.28 ± 0.21^#^	3.59 ± 0.41
Low-salt group	5	355.33 ± 22.12	1180.00 ± 26.85^#^	3.32 ± 0.14^#^	3.32 ± 0.16^#^
High-salt group	5	353.33 ± 25.01	1284.33 ± 73.55^#^	3.64 ± 0.26	3.63 ± 0.42
PTH + low-salt group	5	426.40 ± 27.66	1341.60 ± 206.33	3.15 ± 0.50	3.52 ± 0.53
PTH + high-salt group	5	425.20 ± 35.88	1666.20 ± 176.44^*^	3.97 ± 0.51	4.07 ± 0.33^*^
PTH + low salt + captopril group	4	443.00 ± 43.62	1387.25 ± 100.05	3.15 ± 0.33	3.50 ± 0.05
PTH + high salt + captopril group	5	457.20 ± 37.08	1579.60 ± 197.27	3.50 ± 0.68	3.46 ± 0.51

The impact of PTH and different salt intake concentrations on the morphology of rat cardiac tissue

**Figure 1:**
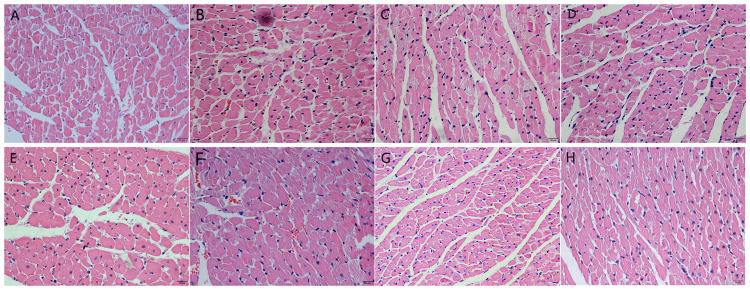
Influence of parathyroid hormone and different salt intake on rat myocardial tissue morphology (magnification ×400) (A) Sham group; (B) PTH group; (C) low-salt group; (D) high-salt group; (E) PTH + low-salt group; (F) PTH + high-salt group; (G) PTH + low salt + captopril group; and (H) PTH + high salt + captopril group. PTH, parathyroid hormone

In the sham group, the cardiomyocytes in the apical part of the left ventricle of the rats were arranged neatly, with clear nuclear staining, uniform cell size, no degeneration or necrosis of the cardiomyocytes, and no inflammatory cell infiltration in the interstitium. Compared with the rest of the groups, the cardiomyocytes in the PTH + high-salt group were significantly hypertrophic and deformed, with disordered arrangement, visible cardiomyocyte dissolution and necrosis, and a large amount of inflammatory cell infiltration. The cardiomyocytes in the PTH + low-salt group were mildly hypertrophic, with a slightly disordered arrangement, and no obvious cardiomyocyte necrosis and inflammatory cell infiltration were observed. After intervention with captopril, the pathological changes in the myocardium caused by the interaction of PTH and high salt can be improved to varying degrees, and the improvement of myocardial lesions in the PTH + low-salt group is more obvious than that in the PTH + high-salt group (Figure 1).

The impact of PTH and different concentrations of salt intake on the expression of myocardial fibrosis marker collagen type III

**Figure 2:**
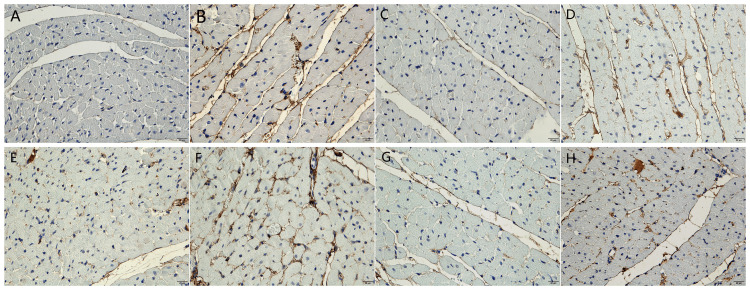
Influence of parathyroid hormone and different salt intake on the expression of type III collagen protein, a marker of myocardial fibrosis (magnification ×400). (A) Sham group; (B) PTH group; (C) low-salt group; (D) high-salt group; (E) PTH + low-salt group; (F) PTH + high-salt group; (G) PTH + low salt + captopril group; (H) PTH + high salt + captopril group. PTH, parathyroid hormone

**Figure 3:**
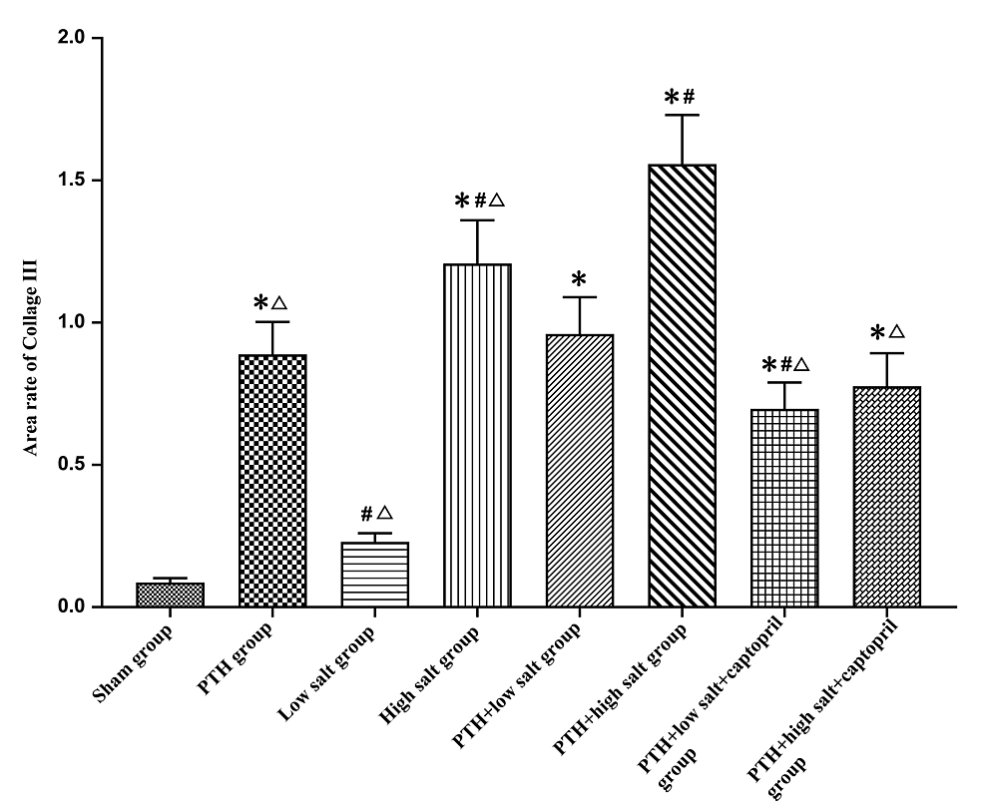
Influence of parathyroid hormone and different salt intake on the expression of myocardial fibrosis marker type III collagen protein. Compared with the sham group: ^*^P < 0.05. Compared with the PTH + low-salt group: ^#^*P *< 0.05. Compared with the PTH + high-salt group: ^△^*P *< 0.05. PTH, parathyroid hormone

In this experiment, we utilized immunohistochemical techniques to detect the expression of type III collagen in myocardial tissue and employed Image-J software (National Institutes of Health) to analyze the positive expression area of type III collagen in each group. The results indicated that, compared to the sham group, the expression of type III collagen significantly increased in all groups except the low-salt group (*P *< 0.05). The positive expression of type III collagen in the PTH + high-salt group was significantly higher than in other groups (*P *< 0.05). The expression level in the PTH + low-salt group was significantly higher than that in the low-salt group and the PTH + low salt + captopril group (*P *< 0.05) (Figures 2-3).

## Discussion

LVH represents the heart's compensatory function, frequently observed in patients with hypertension. This condition instigates myocardial ischemia and triggers myocardial fibrosis and apoptosis of myocardial cells, potentially leading to heart failure. However, it is essential to note that LVH is not exclusively an adaptive response to hypertension [6]. Numerous studies have identified that high sodium intake and elevated levels of PTH are independent risk factors for myocardial hypertrophy, irrespective of their role in blood pressure regulation [7-12].

Prior research has demonstrated that PTH can directly impact myocardial cells, inducing an inward flow of calcium ions and the release of norepinephrine from myocardial cells. This process enhances myocardial contractility and results in the enlargement of myocardial cells, which subsequently leads to LVH and an increase in heart weight [11]. Elevated serum PTH concentrations have also been associated with increased expression of Ang II in the myocardium, contributing to myocardial fibrosis and upregulation of type III collagen protein [10].

LVH and myocardial fibrosis induced by high salt intake are closely linked to the increased expression of Ang II in the RAAS [8]. Based on Dahl's research [13,14] and our findings, we confirmed that high salt intake and PTH operate independently and synergistically to induce LVH. High salt intake increases the excretory burden on the kidneys, leading to increased urinary calcium excretion and a secondary elevation in PTH.

Although previous studies have suggested that PTH may have a protective effect on the hearts of adult rats, it has been found to exert a detrimental impact on the cardiovascular system of spontaneously hypertensive rats (SHR) and elderly rats [15]. Prolonged exposure to PTH can desensitize vascular tissue to PTH, rendering it more susceptible to vasoconstrictors such as Ang II and leading to coronary artery calcification [16,17].

Dahl's animal experiments have confirmed the impact of high salt intake on blood pressure and revealed that a high-salt diet seems only to affect rats genetically predisposed to salt-sensitive hypertension [18,19]. Therefore, when high salt intake or PTH acts independently, it may necessitate high-concentration intervention, long-term exposure, or even the selection of genetically hypertensive rats to potentially demonstrate an increase in blood pressure, LVH, and the occurrence of myocardial fibrosis.

However, our study confirms that high salt intake and PTH can interact in Sprague-Dawley rats to produce a synergistic effect, resulting in pathological changes such as myocardial hypertrophy and myocardial fibrosis in a short period. Our research holds significant clinical value, particularly for individuals with significantly elevated PTH levels in patients with cardiovascular diseases or CKDs. In such cases, a strict salt restriction may aid in reducing the occurrence of LVH and even heart failure.

The cardioprotective efficacy of ACEI has been substantiated by many studies [20,21]. ACEI can reduce blood pressure, ameliorate LVH, and, consequently, enhance the prognosis of cardiovascular diseases. ACEI exerts its effects primarily by acting on the RAAS, inhibiting the production of Ang II and decreasing aldosterone secretion. This cascade of events mitigates water and sodium retention and improves arterial compliance [22]. Additionally, ACEI has been shown to reduce oxidative stress [23], downregulate PTH expression [24], and exhibit anti-fibrotic properties [25].

Further research has unveiled that ACEI demonstrates a greater efficacy in reducing blood pressure and LVH when administered in conjunction with a low-salt diet compared to a high-salt diet [26]. However, our study also revealed that captopril could not completely reverse the LVH induced by the combined PTH and high salt intervention. This finding underscores the importance of stringent salt restriction in patients diagnosed with cardiovascular diseases or CKDs.

This study, while insightful, presents certain limitations that must be acknowledged. These include a relatively small sample size, a limited observation period, and a lack of comprehensive investigation into the underlying mechanisms. As a result, it remains unclear which signaling pathways are involved in the pathophysiological changes associated with LVH. For future research, expanding the sample size and extending the observation period would be beneficial. This would provide a more robust data set and allow for more accurate conclusions to be drawn. Additionally, it would be advantageous to delve deeper into exploring potential influencing factors and thoroughly investigate the signaling pathways. This could shed light on the intricate processes involved in the onset and progression of LVH, thereby contributing to a more comprehensive understanding of this condition.

## Conclusions

The concomitant intervention of PTH and high salt intake can instigate an escalation in blood pressure, LVH, and myocardial fibrosis levels in Sprague-Dawley rats. Introducing captopril as an intervention can mitigate blood pressure and attenuate pathological alterations in myocardial tissue. However, it is incapable of completely reversing LVH. The findings suggest a complex interplay of factors and underscore the need for a multifaceted approach to manage such conditions.
